# Multidimensional Perfectionism and Facial Symmetry, Attractiveness and Approachability: Comparing Those With High Versus Low Dysmorphic Concerns

**DOI:** 10.1177/00332941231205274

**Published:** 2023-10-03

**Authors:** Wei Lin Toh, Sandy Lam, Madeleine Mangano, Susan L. Rossell

**Affiliations:** Centre for Mental Health and Brain Sciences, 3783 Swinburne University of Technology, Melbourne, VIC, Australia; Department of Psychiatry, St Vincent’s Hospital, Melbourne, VIC, Australia; Department of Psychology, Alfred Hospital, Melbourne, VIC, Australia; Centre for Mental Health and Brain Sciences, 3783 Swinburne University of Technology, Melbourne, VIC, Australia; Centre for Mental Health and Brain Sciences, 3783 Swinburne University of Technology, Melbourne, VIC, Australia; Centre for Mental Health and Brain Sciences, 3783 Swinburne University of Technology, Melbourne, VIC, Australia; Department of Psychiatry, St Vincent’s Hospital, Melbourne, VIC, Australia

**Keywords:** Body dysmorphic disorder, perfectionism, face perception, symmetry, attractiveness, approachability

## Abstract

Concerns pertaining to one’s physical appearance or specific body parts is not uncommon in the community. Whether such dissatisfaction is related to superior (or inferior) face perception abilities, or interacts with related constructs, such as perfectionism, is unknown. The current study aimed to investigate whether multidimensional perfectionism (e.g. involving *concern over mistakes* or *doubts over actions*) and facial ratings differed in those with high versus low dysmorphic concerns (i.e. excessive preoccupation about perceived physical flaws). Respondents (*N* = 343) from the community took part in an online study, comprising questionnaires assessing dysmorphic concerns and perfectionism. They also completed a face perception task involving symmetry, attractiveness and approachability ratings for a series of faces, some of which had been digitally manipulated to yield differing degrees of symmetry. Respondents were divided into those with high (*n* = 147) versus low (*n* = 196) dysmorphic concerns. Group comparisons using analyses of variance were conducted. Those with high dysmorphic concerns exhibited significantly elevated overall perfectionism (as well as on facets involving *concern over mistakes*, *personal standards*, *parental perceptions* and *doubts over actions*). No significant group differences were uncovered for the face perception task, involving ratings of symmetry, attractiveness and approachability. Perfectionism differences existed in a non-clinical sample with high dysmorphic concerns, though further work is needed to elucidate consistent patterns regarding perfectionism facets. More research examining face perception deficits on the clinical end of the body image spectrum, such as in those with body dysmorphic disorder, as well as utilising alternate task versions involving self-referential stimuli, are recommended.

## Introduction

Clinically significant dysmorphic concerns affect a segment of the community (Buhlmann et al., 2010), but dissatisfaction with overall physical appearance or disturbances affecting one or more body parts can be quite widespread (Bartsch, 2007; Mond et al., 2013). The clinical end of this spectrum often manifests as body dysmorphic disorder (BDD), where individuals hold an excessive preoccupation with slight or imagined physical flaw(s), and engage in repetitive, time-consuming mental acts or behaviours in an attempt to address or camouflage these (American Psychiatric Association, 2022). One aspect of body dissatisfaction in BDD, as well as non-clinical cohorts, involves appearance-related symmetry concerns, which tend to be focused on facial features (Hart & Phillips, 2013; Silver et al., 2010; Toh et al., 2020). How these symmetry concerns may arise, whether they involve differences in facial perception abilities, or interact with related constructs, such as perfectionism, is unknown. The current study therefore aimed to examine whether multidimensional perfectionism and facial perception differed significantly in a community sample with high versus low dysmorphic concerns.

### Multidimensional Perfectionism

Perfectionism can be broadly characterised as striving to achieve high levels of performance, alongside critical self-evaluations, and is represented by adaptive versus maladaptive facets (Shafran et al., 2002). Those with BDD have reported significantly increased maladaptive perfectionism relative to healthy controls (Schieber et al., 2013). According to the Frost et al. (1990) multidimensional model of perfectionism, six facets exist: C*oncern over mistakes* involves the setting of high personal standards, accompanied by overly self-critical evaluations; *organisation* focuses on order and precision; *personal standards* emphasise individual striving and achievement; *parental expectations* and *criticism* respectively refer to anticipation that familial expectations will be met, and the ensuing loss of acceptance should such goals not be achieved; and *doubts over actions* pertain to misgivings regarding the quality of personal performance. It was found that those with BDD displayed significantly heightened overall perfectionism, as well as *concern over mistakes* and *doubts over actions*, with no significant group differences uncovered for the remaining subscales (Buhlmann et al., 2008). Further investigations into multidimensional perfectionism in non-clinical samples are however warranted.

### Facial Symmetry Ratings

Though the first record of human interest in symmetry may be traced back to the design of Acheulean tools half a million years ago (Hodgson, 2011), symmetry preference has been shown to be the strongest for human faces relative to other forms of visual stimuli, likely owing to a combination of perceptual and evolutionary biases (Little, 2014). That being said, studies into symmetry in BDD have utilised varying stimuli, ranging from geometric objects (Buhlmann et al., 2014), dot probes (Reese et al., 2010), and building images (Lambrou et al., 2011). However, those employing human faces offer the most ecological validity, owing to the fundamental role that accurate face perception plays in facilitating effective communication and social adaptation in everyday life (Cheal & Rutherford, 2011).

Three studies have examined facial symmetry detection in BDD (Buhlmann et al., 2014; Lambrou et al., 2011; Reese et al., 2010), with task variations and mixed findings. The general paradigm involved digitally manipulating facial photographs to yield images with varying levels of symmetry. Respondents then viewed these facial photographs sequentially in pairs, and were asked to either indicate whether each pair was identical or not (Buhlmann et al., 2014), or select which face within each pair was more symmetrical (Reese et al., 2010). In Lambrou et al. (2011), participants’ own facial photographs were used, and they were instead asked to pick out their ‘actual’ facial image from nine sequential presentations (eight of which had been digitally manipulated in terms of symmetry). For two of the studies (Buhlmann et al., 2014; Reese et al., 2010), no significant symmetry detection differences were found between BDD and control groups. However, when trials involving unchanged facial images were exclusively analysed, it was found that the BDD group rated identical facial images as being changed significantly more frequently relative to controls, indicating a tendency for greater perceptual errors (Buhlmann et al., 2014). In Lambrou et al. (2011), participants with BDD were conversely significantly more accurate in perceiving their actual facial image relative to controls. Methodological differences (e.g. minor task variations, use of own vs. others’ facial images) make it challenging to directly compare these findings, or draw definitive conclusions. We may however speculate that symmetry perception in BDD may be enhanced for own face stimuli, but conversely reduced for other faces, although underlying reasons for this remain unknown.

### Facial Attractiveness and Approachability Ratings

Other than symmetry, examining facial attractiveness and approachability may be pertinent in relation to dysmorphic concerns. This is because predominant concerns are known to centre on the face for many individuals with BDD (Toh et al., 2020). Moreover, processes of social comparison and feedback mean that those with the disorder have been theorised to misjudge interactions with others as hostile owing to their perceived physical flaws, leading to fears involving lack of social desirability and subsequent rejection (Buhlmann et al., 2004). In fact, high levels of comorbid social anxiety are known to exist in BDD (Fang & Hofmann, 2010).

Several studies have investigated facial attractiveness ratings in BDD, where it was generally found that those with the disorder tended to overstate the attractiveness of others’ faces, whilst understating their own facial attractiveness (Buhlmann et al., 2008; Lambrou et al., 2011). Participants with BDD also tended to rate symmetrical faces as more attractive (Lambrou et al., 2011; Reese et al., 2010). There has conversely been a lack of research regarding approachability judgements in BDD, but given fears of social rejection coupled with elevated social anxiety (Buhlmann et al., 2004; Fang & Hofmann, 2010), further exploration of this construct is worthwhile.

### Aims and Hypotheses

The current study aimed to compare perfectionism as well as facial symmetry, attractiveness and approachability ratings in those with high versus low dysmorphic concerns. Employing a community sample was deemed appropriate as these constructs vary dimensionally, and their involvement helps to circumvent prominent issues in clinical studies, including small participant numbers, high psychiatric comorbidities and confounding medication effects.

For multidimensional perfectionism, it was hypothesised that those with high dysmorphic concerns would endorse significantly greater overall perfectionism as well as in facets involving *concern over mistakes* and *doubts over actions* relative to those with low dysmorphic concerns (Buhlmann et al., 2008). Due to mixed evidence (Buhlmann et al., 2014; Lambrou et al., 2011; Reese et al., 2010), it was uncertain whether those with high dysmorphic concerns would be more, less or equally accurate in facial symmetry detection relative to those with low dysmorphic concerns; no specific hypothesis was offered in this regard. Similarly, our analysis on attractiveness and approachability ratings were exploratory. Finally, we sought to explore the interrelations amongst perfectionism levels, facial ratings and level of dysmorphic concerns, where we tentatively expected a significant positive correlation between overall perfectionism and dysmorphic concerns; patterns of associations with facial ratings remained exploratory owing to conflicting empirical findings. Should significant associations with dysmorphic concerns be established, post-hoc regressions may be employed to further explore the potential contributions of multidimensional perfectionism and overall face perception.

## Method

### Participants and Procedure

Respondents were 444 individuals recruited from two primary sources – the general community (*n* = 192) based on advertising on research participation forums (e.g. Gumtree) and social media (e.g. Facebook), as well as first-year Psychology students (*n* = 252) enrolled in the Research Experience Program (REP) at Swinburne University of Technology. Eligibility criteria comprised: (i) being aged 18 years or above, (ii) sufficient English language proficiency to respond meaningfully to survey questions, and (iii) availability of a laptop or tablet device with internet connectivity (use of mobile phones was not permitted due to insufficient screen resolution for the face perception task, as stipulated in the study instructions).

Participation involved undertaking an hour-long online survey via Qualtrics (mean completion time = 53.1 ± 26.8mins), and respondents were financially reimbursed (or allocated research participant credits). Stringent data cleaning was undertaken, where respondents who: (i) completed less than 50% of the total survey (*n* = 69), (ii) had a completion time more than 1.5 standard deviations below the mean, to eliminate random, spurious responding (*n* = 24), or (iii) contravened task instructions by using a mobile phone (*n* = 8), were excluded. The final dataset comprised of 343 individuals. The study was approved by the Swinburne University of Technology Human Research Ethics Committee (#20200664-4153), and procedures conformed to the Declaration of Helsinki (World Medical, 2013). Respondents also provided informed consent, and were provided with a debriefing statement upon study completion.

### Measures

Basic sociodemographic (e.g. age, sex, education) and mental health information (i.e. self-report psychiatric diagnosis, psychological treatment) were collected. Negative emotions were evaluated using the Depression Anxiety Stress Scales (DASS-21; Lovibond & Lovibond, 1995). This abbreviated 21-item measure aims to assess negative emotions, including depression, anxiety and stress, rated on four-point Likert scales. Scores within the three subscales are summed, with higher scores denoting increased negative emotions; a total score can also be obtained by further summing subscale scores. Dysmorphic concerns were assessed using the Dysmorphic Concerns Questionnaire (DCQ; Oosthuizen et al., 1998). This seven-item measure aims to assess appearance-related concerns, rated on four-point Likert scales. These are summed to yield a total score, with higher scores denoting elevated dysmorphic concerns. Perfectionism was evaluated using the Frost Multidimensional Perfectionism Scale – short form (FMPS-sf; Cox et al., 2002). This abbreviated 22-item measure aims to assess five facets of perfectionism, namely *concern over mistakes*, *organisation*, *personal standards*, *parental perceptions* and *doubts over actions*, rated on five-point Likert scales (nb. two facets relating to *parental expectations* and *criticisms* in the original measure were amalgamated into a single factor represented by *parental perceptions* in this version). Scores within the five subscales are summed, with higher scores denoting increased perfectionism; a total score can also be obtained by further summing subscale scores.

The face perception task was adapted from Rhodes et al. (1998), with three modifications: (i) 20 (instead of 48) facial images were utilised, (ii) a low symmetry variant (i.e. 50% increase between original and perfect symmetry faces) was omitted, and (iii) an approachability rating was introduced (in lieu of a mate appeal rating for opposite-sex faces only). Our task thus aimed to assess respondents’ perceptions of facial symmetry, attractiveness, and approachability. Twenty coloured facial photographs bearing neutral expressions (10 male, 10 female), were selected from the Chicago face database (Ma et al., 2015), to reflect the approximate demographic distribution in terms of age, sex and ethnicity of the Australian general population (Australian Bureau of Statistics, 2016). These original facial images were digitally manipulated using Abrosoft Fantamorph (at 50% morph) and Adobe Photoshop to create versions representing increasing facial symmetry (see Appendix A). Three symmetry variants (i.e. veridical, high and perfect symmetry) were generated (1537 x 1080 pixels). These 60 trials were sequentially presented in pseudorandomised order (preceded by practice trials). For each face, respondents were asked to rate symmetry, attractiveness, and approachability, ranging from *0 = Not at all* to *10 = Extremely*. There was no time limit and latencies were not recorded.

### Data Analysis

Data analysis was performed with IBM SPSS, v.27. Respondents were divided into two subgroups depending on their endorsed level of dysmorphic concerns, using a conservative cut-off score of ≥14 on the DCQ (Stangier et al., 2003; see Table 1). To characterise our subgroups, preliminary analysis involved group-wise comparisons of demographic and clinical information. One-way analyses of variances (ANOVAs) were conducted for continuous variables. For violations of homoscedasticity, Welch’s *F* was reported (Field, 2009). Bonferroni corrections were applied, where appropriate. For categorical variables, chi-squared tests for independence were employed. To test our hypothesis, group-wise comparisons were conducted between the high versus low DCQ groups on total and dimensional perfectionism. For our exploratory analyses, a mixed between-within analysis of variance (ANOVA) was conducted based on the face perception task, with facial ratings as the main outcome variable, group as the between-subjects factor, task (i.e. symmetry, attractiveness and approachability) as the first within-subjects factor, and symmetry manipulation (i.e. veridical, high and perfect) as the second within-subjects factor.

**Table 1.** Demographic and Clinical Information of Low Versus High DCQ Groups.

	Means ± standard deviations or percentages		Statistics^a^		
	*Low DCQ (n = 196)*	*High DCQ (n = 147)*	F or χ^2^	*p*	η^2^ or φ
Demographic					
Age (years)	32.7 ± 10.9	29.8 ± 9.9	7.0^b^	**.009**	.020
Sex (% male)	24.2	23.0	1.2	.527	.062
Education (years)	14.1 ± 2.4	14.0 ± 2.5	0.2	.626	.001
Employment (% employed)	67.6	71.6	0.5	.502	.044
Marital status (% partnered)	48.9	43.9	0.6	.429	.050
Clinical					
Psychiatric diagnosis (% yes)	15.4	30.4	9.8	**.002**	.180
Psychiatric medication (% yes)	3.3	14.9	12.6	**<.001**	.206
Psychological treatment (% yes)	85.7	93.3	0.4	.505	.126
Dysmorphic concerns (DCQ)	9.7 ± 1.9	18.0 ± 3.3	724.7^b^	**<.001**	.710
Negative emotions (DASS-21)	10.9 ± 10.3	25.0 ± 12.6	122.8^b^	**<.001**	.276
Depression	3.0 ± 3.7	8.0 ± 5.1	100.3^b^	**<.001**	.243
Anxiety	2.7 ± 3.4	7.0 ± 4.6	90.0^b^	**<.001**	.223
Stress	5.2 ± 4.8	10.0 ± 4.8	91.0	**<.001**	.211
Other					
Perfectionism (FMPS-sf)	63.6 ± 14.7	75.6 ± 11.3	72.6^b^	**<.001**	.165
Concern over mistakes	11.9 ± 4.9	16.4 ± 4.3	77.8	**<.001**	.186
Organisation	14.6 ± 3.3	14.6 ± 3.1	<0.1	.918	<.001
Personal standards	16.4 ± 4.7	18.7 ± 3.4	26.6^b^	**<.001**	.067
Parental perceptions	12.6 ± 5.4	15.6 ± 4.9	27.2	**<.001**	.074
Doubts over actions	8.1 ± 2.9	10.4 ± 2.3	66.8^b^	**<.001**	.155

*Note.* DCQ = Dysmorphic Concerns Questionnaire; DASS-21 = Depression Anxiety and Stress Scale; FMPS-sf = Frost Multidimensional Perfectionism Scale-short form; Bonferroni correction (DASS-21 subscales = .05/3 = .017; FMPS-sf subscales = .05/5 = .01).

^a^One-way ANOVAs with effect size ƞ^2^: .01 = small, .06 = medium, .14 = large; φ: .10 = small, .30 = medium, .50 = large.

^b^Based on Welch’s *F*.

## Results

Group-wise comparisons of demographic and clinical information as well as multidimensional perfectionism are shown in Table 1. The high DCQ group was significantly younger than the low DCQ group; no other significant demographic differences were found. In terms of clinical variables, a significantly greater proportion of the high DCQ group had received a psychiatric diagnosis and/or medication, but there were no significant group differences in terms of psychological treatment. The high DCQ group was also significantly more depressed, anxious and stressed. Notably, the high DCQ group exhibited significantly elevated overall perfectionism compared to the low DCQ group. On a dimensional level, this translated to the high DCQ group showing significantly greater perfectionism on all facets, except for *organisation*, where no significant group differences were found.

**Figure 1.**
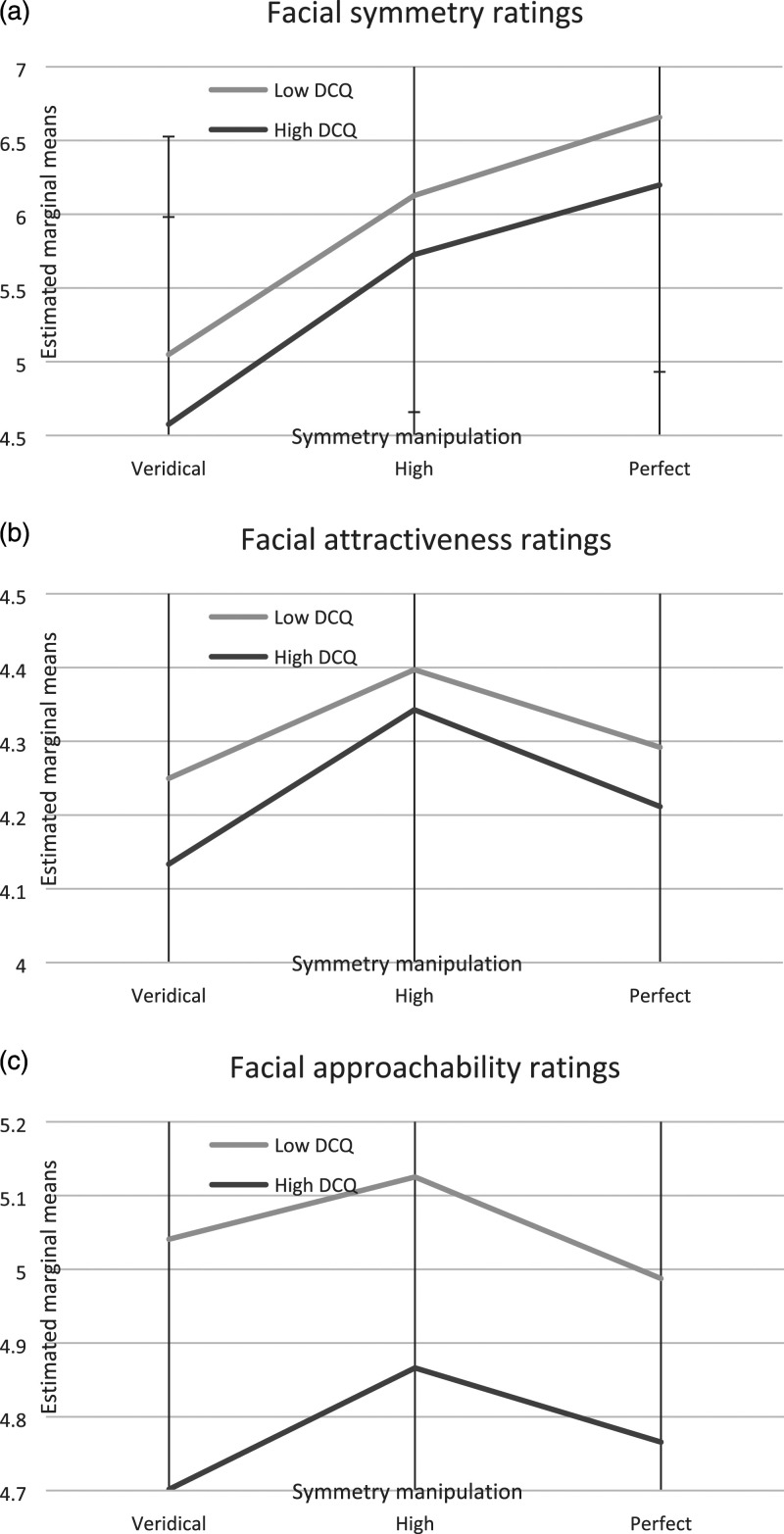
Estimated marginal means (with error bars) for low versus high DCQ groups in relation to (a) facial symmetry ratings, (b) facial attractiveness ratings, and (c) facial approachability ratings.

For the face perception task, a mixed between-within ANOVA was conducted, with results shown in Table 2. Interactions involving group by task, group by symmetry manipulation, and group by task by symmetry manipulation were not significant (though the task by symmetry manipulation interaction was significant). The main effect of task was significant; the entire sample rated faces as more symmetrical (*M* = 5.8, *SE* = .1) than attractive (*M* = 4.3, *SE* = .1) or approachable (*M* = 4.9, *SE* = .1). The main effect of symmetry manipulation was also significant; perfect symmetry faces (*M* = 6.5, *SE* = .1) were given higher symmetry ratings than high symmetry (*M* = 6.0, *SE* = .1) or veridical (*M* = 4.8, *SE* = .1) faces. The main effect comparing groups was significant, with the high DCQ group (*M* = 4.8, *SE* = .1) significantly more critical in their ratings throughout relative to the low DCQ group (*M* = 5.1, *SE* = .1). Estimated marginal means, with accompanying standard deviations, for the three task conditions (i.e. symmetry, attractiveness and approachability) by group are shown in Figure 1 (noting that only the main effects, but not any of the interactions, were significant). From this, we can observe that the high DCQ group consistently provided lower facial symmetry, attractiveness and approachability ratings throughout regardless of symmetry manipulation, with high symmetry (relative to veridical or perfect symmetry) faces preferred by both groups in terms of greatest attractiveness and approachability.

**Table 2.** Mixed Between-Within ANOVA for Face Perception Task (*N* = 336).

	*F*	*p*	*Partial η* ^*2*^	*Contrasts*
Group	5.9	**.046**	.011	-
Task	166.9	**<.001**	.5.1	Symmetry > Attractiveness; Symmetry > Approachability
Symmetry manipulation	237.1	**<.001**	.587	Perfect > Veridical; Perfect > High
Group x task	2.7	.068	.016	-
Group x symmetry manipulation	1.3	.267	.008	-
Task x symmetry manipulation	94.3	**<.001**	.533	-
Group x task x symmetry manipulation	1.3	.245	.016	-

Finally, overall perfectionism was significantly positively correlated with level of dysmorphic concerns (see Table 3). No significant correlations were found with facial ratings (though symmetry, attractiveness and approachability ratings were significantly intercorrelated). Owing to the lack of association between facial ratings and dysmorphic concerns, no further analysis was conducted.

**Table 3.** Pearson Correlation Analysis Amongst Dysmorphic Concerns, Perfectionism and Facial Ratings (*N* = 343).

	Dysmorphic concerns (DCQ)	Perfectionism total (FMPS-sf)	Symmetry ratings	Attractiveness ratings
**Dysmorphic concerns (DCQ)**	1			
**Perfectionism total (FMPS-sf)**	.453**	1		
**Symmetry ratings**	−.049	−.083	1	
**Attractiveness ratings**	.005	.019	.513**	1
**Approachability ratings**	−.018	.019	.613**	.749**

*Note.* DCQ = Dysmorphic Concerns Questionnaire; FMPS-sf = Frost Multidimensional Perfectionism Scale-short form.

*p < .05; **p < .01.

## Discussion

The current study aimed to compare whether individuals with high dysmorphic concerns performed significantly differently to those with low dysmorphic concerns on measures assessing multidimensional perfectionism and ratings of facial symmetry, attractiveness, and approachability. Our two subgroups were relatively well-matched in terms of demographics, except that the high DCQ group was significantly younger than the low DCQ group (though age did not correlate significantly with any of the main outcome variables; *r* = −.008–.123). Several clinical differences did emerge, with the high DCQ group endorsing significantly more psychiatric diagnoses/medications, and reporting significantly greater negative emotions than the low DCQ group, which was perhaps to be expected.

Our hypothesis was fully supported; the high DCQ group endorsed significantly greater overall perfectionism as well as in facets involving *concern over mistakes* and *doubts over actions* relative to the low DCQ group. No significant group differences were found for *organisation*, which was expected, as this facet has been acknowledged to not load as soundly onto the model (Cox et al., 2002; Frost et al., 1990). Notably, the high DCQ group showed significantly heightened *personal standards* and *parental perceptions* relative to the low DCQ group. These findings were contrary to those reported in Buhlmann et al. (2008), where group differences were not found. Our findings instead concur with some existing community studies in perfectionism, based on an alternate Hewitt and Flett (1991) model, where facets are defined by the target to or from whom the perfectionism is ascribed; *self-* and *other-oriented* perfectionism encompass perfectionistic standards targeted at the self and other respectively, whereas *socially prescribed* perfectionism stems from collective societal expectations. Accordingly, *self-oriented* and *socially-prescribed*, but not *other-oriented*, perfectionism are known to significantly predict dysmorphic concerns in the general population (Bartsch, 2007; Cunningham et al., 2018; Hanstock & O'Mahony, 2002). These facets may be respectively theorised as fairly akin to the *personal standards* and *parental perceptions* dimensions in our study. The involvement of different cohorts and varying operationalisations of perfectionism could be partially liable for these discrepant findings, but it remains unclear why the influence of these facets is absent on the clinical end of the spectrum (Buhlmann et al., 2008), but arises in non-clinical groups. Further research is thus warranted.

In our exploratory mixed between-within ANOVA, all of the group-wise interaction terms were not significant. This is perhaps not unexpected, given most existing literature documenting clear differences with regards to symmetry ratings only when stimuli involving participants’ own faces were employed (Buhlmann et al., 2008; Lambrou et al., 2011). Moreover, those studies were primarily conducted in clinical cohorts experiencing BDD symptoms, whereas our study employed a community sample with varying levels of dysmorphic concerns. Yet our findings may not preclude the existence of anomalies in facial judgements. One possibility could be that subtleties underlying these potential deficits means they likely only arise in conjunction with self-referential stimuli (i.e. own faces) and/or in the context of BDD, rather than subclinically. Moreover, our complex task design, incorporating attractiveness and approachability ratings resulted in reduced power associated with introducing a mixed model. That being said, there is merit in examining these related constructs, given their pertinence to BDD symptoms and illness behaviours (e.g. social fears of judgment and negative evaluation). Added investigations to clarify the operation of these constructs and their potential interactions are needed. A deeper understanding of potential multifarious interactions amongst multidimensional perfectionism, facial perception and dysmorphic concerns in general population versus clinical cohorts will not only aid in delineating differences in those with and without BDD, but could also convey important therapeutic implications. In particular, clearly identified deficits (e.g. perfectionism facets impaired in clinical groups, but preserved in non-clinical groups) may serve as viable therapeutic targets around which new therapies may be developed, or existing therapies be adapted to address.

### Limitations

There were several limitations and strengths associated with the current study. Its online nature and lack of face-to-face contact to corroborate participant eligibility and task instructions was one drawback. However, stringent data cleaning procedures were conducted to minimise the possibility of random, erroneous responding. Approximately half of recruited respondents was made up of first-year Psychology students, and three-quarters were female. These demographic skews may have rendered our findings less generalisable. Furthermore, collection of participants’ racial demographics was omitted, meaning we were unable to further analyse potential effects on facial ratings, though we did ensure that employed faces were reflective of the ethnic diversity in the Australian general population. That being said, student cohorts are known to exhibit high levels of dysmorphic concerns (Bartsch, 2007), making involvement of this group particularly appropriate for the purposes of our study. In addition, our robust respondent numbers and use of validated measures, including an alternate conceptualisation of multidimensional perfectionism (Frost et al., 1990), as well as an update to the face perception task (Rhodes et al., 1998) comprising demographically representative, high-resolution facial stimuli serve as key strengths.

### Future Research

There are a number of constructive avenues future research may extend to. These studies need to be expanded to comprise clinical cohorts involving BDD, to ascertain which of the nascent findings also apply to those on the clinical end of the body image continuum. To this end, a lack of significant findings in community studies should not be taken as evidence that potential deficits do not exist, as these can be subtle and potentially arise only in the context of severe BDD. In terms of symmetry ratings, future studies could even focus on BDD subgroups, particularly those who have endorsed predominant symmetry preoccupations, whether centred on the face or other body parts. More nuanced investigation of approachability ratings in the disorder are also worthwhile, given prominent links to social anxiety and rejection in BDD (Buhlmann et al., 2004; Fang & Hofmann, 2010). Related to this, differing versions of the face perception task should be employed, which can variously assess absolute as well as relative (to clinical and non-clinical groups) accuracy of facial judgements. In particular, employing one’s own facial images (where possible) may be especially pertinent. Taking into account the influence of possible biases in recognising transracial faces would also form an added element for consideration in designing future studies (Singh et al., 2022) Given tenuous associations between perfectionism and facial ratings, examination of other possible underlying mechanisms would be fruitful. This can involve top-down or bottom-up processes, respectively involving aesthetic sensitivity or basic visual processing deficits, for instance.

The current study aimed to conduct group-wise comparisons in multidimensional perfectionism and facial ratings involving symmetry, attractiveness and approachability in relation to dysmorphic concerns. Those with high dysmorphic concerns exhibited significantly elevated perfectionism across most facets, though no significant group-wise findings were uncovered with regards to our face perception task. Nevertheless, a general trend was noted in that those with high dysmorphic concerns consistently rated faces as less symmetrical, attractive and approachable relative to those with low dysmorphic concerns. Future research should seek robust clinical replication in BDD cohorts, including symptom-specific subgroups, and employing alternate versions of the face perception task.

## Supplemental Material

Supplemental Material - Multidimensional Perfectionism and Facial Symmetry, Attractiveness and Approachability: Comparing Those With High Versus Low Dysmorphic Concerns Supplemental Material for Multidimensional Perfectionism and Facial Symmetry, Attractiveness and Approachability: Comparing Those With High Versus Low Dysmorphic Concerns by Wei Lin Toh, Sandy Lam, Madeleine Mangano, and Susan L. Rossell in Psychological Reports

## Data Availability

The dataset is available on request by qualified researchers-scientists. Requests require a concept proposal describing the purpose of data access, appropriate ethics approval, and provision for data security. All data analysis scripts and results files are available for review.
