# Supplementary material for: Multidimensional Perfectionism and Facial Symmetry, Attractiveness and Approachability: Comparing Those With High Versus Low Dysmorphic Concerns
Source: Psychol Rep. 2023 Oct 3;128(5):3441–56. doi: 10.1177/00332941231205274 (PMC12394769; doi:10.1177/00332941231205274)
Supplement: Supplemental Material - Multidimensional Perfectionism and Facial Symmetry, Attractiveness and Approachability: Comparing Those With High Versus Low Dysmorphic Concerns [file sj-pdf-1-prx-10.1177_00332941231205274.pdf]

Appendix A

Creation and selection of high and perfect symmetry faces

**Step 1: Perfect symmetry faces using Adobe Photoshop**
1. Duplicate a layer of the veridical facial image, and use cutting tool to section into vertical halves<br>2. Bring up "refine edge" window, and change "output to" setting to "new layer with layer mask"<br>3. Use "control+T" to transform, and right-click to select "flip horizontal"<br>4. Use arrow keys to adjust until features on layer of cut image match other half of face<br>5. Set opacity to ~60% and rotate where necessary, pressing "enter" when ready<br>6. Select layer mask and bring up brush tool (in black)<br>7. Brush around hair and neck/chest area to reveal layer underneath for more "natural" look; decrease brush size and apply where appropriate to smooth out facial features<br>8. Save final image in jpeg format when ready
**Step 2: Pilot study to select perfect symmetry composite**
Two perfect symmetry composites of each face (left-left and right-right) were created as in Step 1. A pilot study involving healthy volunteers (*N*=100) was conducted, where respondents were presented with a pair of similar perfect symmetry faces, and asked to select which one of the two faces looked more "natural". The version with the higher mean score was selected for inclusion in the face perception task. According to the Chicago Face Database (Ma et al., 2015) identifiers, the faces employed were WM-009 (right), WM-004 (right), WM-201 (left), WM-228 (left), WM-257 (right), LM-224 (left), AM-218 (left), AM-233 (left), AM-226 (left), BM-043 (right), AF-213 (right), AF-235 (right), AF-244 (right), LF-249 (right), WF-002 (left), WF-216 (right), WF-228 (left), WF-240 (left), WF-251 (left).
**Step 3: High symmetry faces using Abrosoft Fantamorph**
1. Import veridical image as Image 1<br>2. Import perfect symmetry image as Image 2<br>3. Set top sliding bar to 50% to "preview"<br>4. Click "add-ins" and "face locator", then "apply" for both Images 1 and 2 in turn<br>5. A total of 112 key dots will be placed on each face, which correspond to each other<br>6. Check the preview image for any blurring, and manually adjust the key dots to minimise<br>7. Manually add key dots to the neck/chest area to ensure smooth morphing<br>8. Click on the camera icon on the right of the preview image to export final image in jpeg format

Example images comprising veridical, perfect symmetry and high symmetry faces

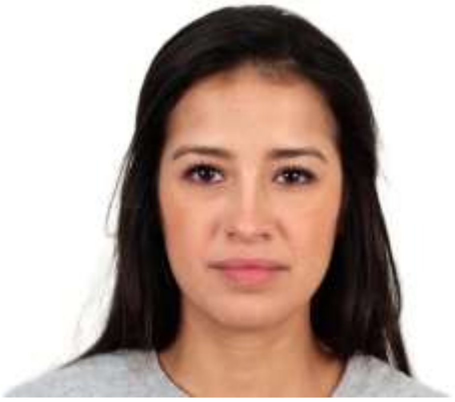

A. Veridical

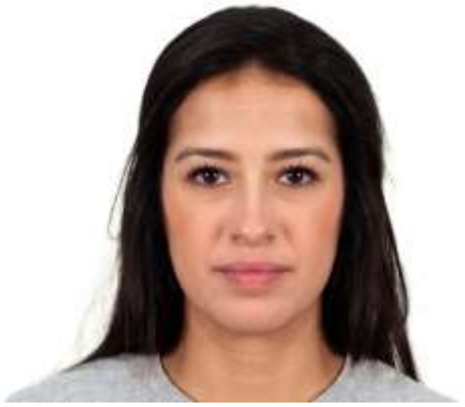

B. Perfect symmetry

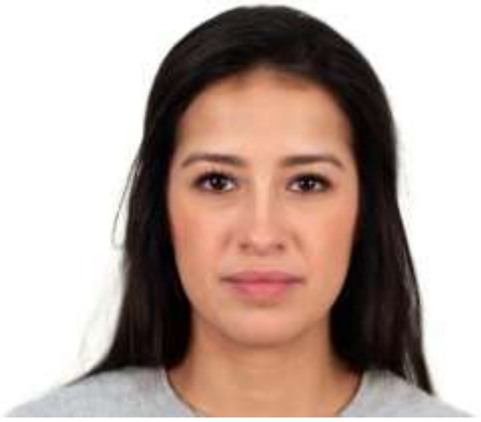

C. High symmetry
